# Umbilical and Ventral Entero-epiplocele, with a Report of Three Cases Successfully Treated

**Published:** 1887-04

**Authors:** Daniel D. Lee

**Affiliations:** Instructor in Anatomy and Operative Veterinary Surgery, Harvard University


### Art. XVII.—UMBILICAL AND VENTRAL ENTERO-EPIPLOCELE, WITH A REPORT OF THREE CASES SUCCESSFULLY TREATED.

BY DANIEL D. LEE, M. D. V.
*Instructor in Anatomy and Operative Veterinary Surgery, Harvard University.*

In the human embryo complete closure of the abdominal cavity takes place at the eighth week. It is not uncommon for a cleft to remain near the umbilicus, through which comes a peritoneal protrusion containing a coil of intestine. This forms a bulging mass, from the apex of which arises the umbilical cord. This is a common malformation. The sac is usually small; in rarer cases the cleft extends for nearly the whole length of the abdominal wall. Occasionally, the abdominal wall is closed in the neighborhood of the umbilicus, while there is a fissure above or below it; in these cases there is generally ecstrophia of the bladder.

These remarks apply equally well to all our animals, the time of closure of the abdominal cavity of course varying with the length of pregnancy. Such herniæ may be recovered from spontaneously during youth by the proportional shortening of the mesentery with the growth of the animal; or they may persist, grow larger, and finally cause the death of the animal, as seen in pigs thus affected, the animal being found dead some morning from strangulated hernia after being fattened for the market. These herniæ may also arise from some external stretching force, by which the peritoneum is dragged outwards, or, it may be pressed outwards by a tumor.

Other causes of the acquired form are degeneration and giving away of the abdominal wall, stretching apart of the fibres of the linea alba, sudden strain or exertion generally causing it. Again I have seen rupture of the muscular walls, the skin remaining entire, caused by a thrust of a cow's horn. The intestines escaped through the opening, and formed a large bulging tumor under the skin. The cow was killed.

To accomplish a cure, I advise an operation which I shall describe. Trusses are useless in all animals, as they are always misplaced or torn off by the efforts of the animal to free itself.

CASE I. A colt about five months old had a tumor over the seat of the umbilicus. I was called by another veterinarian to advise whether it should be operated upon or not. On examination I found the tumor to be an entero-epiplocele, entirely reducible by manipulation, the opening through which the descent took place being about the size of the middle finger. On inquiry I found that the tumor was congenital, but that its size and the capacity of the opening into the abdominal cavity had decreased one-half within the last six weeks. I therefore advised that it be let alone, as the proportional shortening of the mesentery with the growth of the animal would affect a cure. Within a few weeks the tumor had disappeared.

CASE II. A pig four months old, had a small tumor on the belly when bought, but within the last week or two the tumor had so increased in size as to drag on the ground. On examination I found a tumor about the size of a musk melon, which was easily reduced when the pig was laid on his back, but when he walked it dragged on the ground. The opening of the sheath was on the inferior surface of the tumor. I considered the case as needing instant attention, so I ordered the pig starved for two days and gave him a cathartic.

On the third day I secured him on his back, and having etherized him and washed the tumor, I cut through the skin covering the anterior face of the tumor, in order not to injure the sheath, and exposed the peritoneal sac. This I separated by means of my finger, from the skin until I got near the opening in the muscular walls, when I was obliged to use the knife. Having completed the separation, I returned the sac through the abdominal opening and took two wire and three silk stitches through the muscular walls, in order to close the opening. I then sewed up the skin and let the pig go.

Recovery was complete; during the first few days there was considerable serous infiltration into the loose skin over the seat of the tumor; this soon disappeared. I did not take out any of the stitches.

CASE III. The last case was in a dog. The tumor was very small in the beginning but increased till a swelling as large as an egg was seen over the umbilicus. I operated in the same way, except that, the sheath not coming so far forward, I was able to cut on the inferior surface. This case also made a perfect recovery.

I have seen three calves with umbilical entero-epiplocele, who were operated on by the method of ligating the skin after reducing the tumor by manipulation; none of them were benefited. I have also seen a bitch with *ventral entero-epiplocele*, who was operated upon as I recommended, and made a perfect recovery. If it is possible to operate on dogs and pigs, why not on foals and calves?

When a cure is attempted by ligating the skin at the base of the tumor there is but a small chance that the lips of the muscular opening will be brought together, and this is necessary in order to bring about closure of the orifice.

In those cases where the hernia, be it umbilical or ventral, is congenital and does not increase in size, or decrease, I advise letting it alone. In cases where it is acquired or congenital, and *increases* in size, I advise an operation as follows:

Starve the animal for two days, and give a cathartic; give no water for twenty-four hours before operating. Securely restrain the animal, etherize and then turn on the back. Wash the belly thoroughly with soap and water. Reduce the tumor as far as possible by manipulation. Cut through the skin down to the peritoneal sac, separate the sac from the skin and dissect away its adhesions to the lips of the opening in the muscular walls. Wash the sac, and return it and its contents to the abdominal cavity, enlarging the opening in the muscular walls, if necessary, to accomplish this. Take a sufficient number of silk, or wire sutures in the lips of the muscular opening to close it entirely, and suture the skin over this, leaving a depending opening for drainage. Through this opening bring the ends of the inner silk sutures to keep it clear, tie these ends to one of the sutures in the skin. Let the animal go, and keep the parts clean; avoid all severe exercise for a week or more. Take the stitches out or leave them in as you think best, I always do the last.

